# P-1533. In silico Exploration of Cannabidiol Interactions with Outer Membrane Proteins in Salmonella Typhimurium LT2

**DOI:** 10.1093/ofid/ofaf695.1714

**Published:** 2026-01-11

**Authors:** Ibrahim Iddrisu, Emmanuel Ndezure, Junhuan Xu, Robertson k Boakai, Olufemi S Ajayi, Joseph Ayariga

**Affiliations:** Alabama State University, Montgomery, AL; Kwame Nkrumah University of Science and Technology, Kumasi, Ashanti, Ghana; Alabama State University, Montgomery, AL; Alabama State University, Montgomery, AL; Alabama State University, Montgomery, AL; Alabama State University, Montgomery, AL

## Abstract

**Background:**

Cannabidiol (CBD), the non-psychoactive component of the hemp plant has an enormous potential as a novel antimicrobial agent. Several studies have explored CBD’s potential as a potent alternative antimicrobial agent against both Gram-Negative and Gram-Positive pathogens. However, the exact mechanisms or specific interactions between CBD and the membrane is poorly understood.

**Methods:**

This study aimed at understanding the interactions between CBD and the outer membrane proteins (OMPs) of *Salmonella* Typhimurium LT2. Employing *in silico* techniques, we analyzed the binding affinities, interaction dynamics, and drug-likeness of CBD with key OMPs such as OmpA, OmpC, OmpD, OmpF, OmpX, and NompC.

**Results:**

The molecular docking results showed that CBD exhibits varying binding affinities across the OMPs. OmpX and NompC exhibited the highest binding affinity with CBD at -6.6 kcal/mol and -6.4 kcal/mol respectively. The results also revealed several key interactions such as conventional hydrogen bonds, π-stacking, and hydrophobic interactions which plays crucial roles in the stability of the binding complex. We also included phylogenetic analysis of fifty different strains of *S.* Typhimurium, and observed high sequence conservation levels among the OMPs, at a sequence similarity threshold of 90%. This high conservation underscores the importance of targeting the conserved regions for the efficient use of CBD as a broad-spectrum antibiotic.

**Conclusion:**

This may enhance the efficacy of existing antimicrobial treatments. In conclusion, the *in silico* findings suggest that CBD, through its interaction with critical OMPs, has the potential to serve as a potent antimicrobial agent against *S.* Typhimurium LT2. Especially when CBD is formulated to target the conserved regions of the OMPs. These findings are preliminary with several limitations. *In vitro* and *in vivo* studies may be required to validate the findings in this study, laying the foundation for further studies on CBD as a novel therapeutic agent in combating bacterial infections and addressing the global challenge of antibiotic resistance.

**Disclosures:**

All Authors: No reported disclosures

